# Cardiac sarcoidosis: Two case reports

**DOI:** 10.1002/ccr3.4270

**Published:** 2021-06-23

**Authors:** Jane Sandra Afriyie‐Mensah, Felix Razak Awindaogo, Emmanuella Naa Deeidei Tagoe, Harold Ayetey

**Affiliations:** ^1^ Department of Medicine and Therapeutics University of Ghana Medical School Accra Ghana; ^2^ Department of Medicine and Therapeutics Korle Bu Teaching Hospital Accra Ghana; ^3^ Department of Internal Medicine and Therapeutics University of Cape Coast Cape Coast Ghana

**Keywords:** bradycardia, bundle branch block, heart block, heart failure, sarcoidosis

## Abstract

The clinical presentation of cardiac sarcoidosis is variable. We report two cases of cardiac sarcoidosis to highlight the varied clinical presentations and diagnostic challenges in our setting, and encourage the consideration of sarcoidosis as a differential in unexplained arrhythmias and heart failure.

## Graphical abstract

Cardiac sarcoidosis is a major cause of death and disability in sarcoid patients. It can be benign and asymptomatic or life‐threatening. Early diagnosis is lifesaving since prompt therapy can lead to the resolution of cardiac dysfunction.

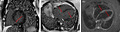

## 1 INTRODUCTION

Sarcoidosis is a multisystem inflammatory disease characterized by the presence of noncaseating granulomas of unknown etiology. Although it affects almost every organ system in the body, lung involvement occurs in over 90% of all cases.^1^ Estimated prevalence of sarcoidosis is 20‐60 cases/100 000 with worse outcomes among African American populations.^1^ There is a lack of data on the incidence and disease burden of sarcoidosis in Africa and Ghana. Detailed clinical and epidemiological studies are urgently required in this area.

Sarcoidosis has the worst prognosis when it involves the heart muscle, accounting for more than two‐thirds of global deaths in sarcoid patients.^2^, ^3^, ^4^ Myocardium involvement may be isolated or occur in association with other organ involvement, particularly pulmonary sarcoidosis.

Clinical presentation of cardiac sarcoidosis (CS) is variable being symptomatic, benign, or life‐threatening with symptoms of heart failure, cardiac arrhythmias, or sudden cardiac death (SCD).^5^ The latter is the most common cause of death from CS and could be the first and only manifestation of CS in some cases.^6^

Diagnosis of CS remains a challenge because of nonspecific symptoms and the absence of a single examination technique to aid the diagnosis. Screening of sarcoid patients is therefore imperative since treatment significantly reduces risk of sudden death.^7^

Management of CS is multidisciplinary involving cardiologists, pulmonologists, radiologists, rheumatologists, and others. The use of immunosuppressive medications and cardiac‐specific therapies to manage cardiac sequelae of sarcoidosis such as heart failure and rhythm disturbances is employed.^8^, ^9^

We present two cases of cardiac sarcoidosis with differing clinical presentations.

## 2 CASE REPORTS

### 2.1 Case 1

**FIGURE 1:**
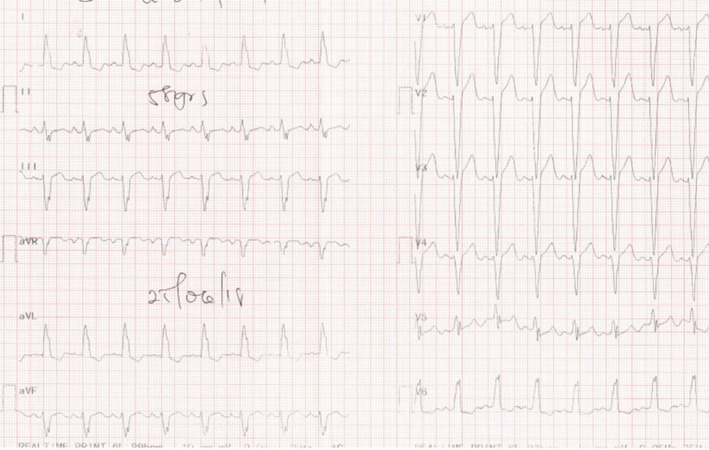
Sinus tachycardia and left bundle branch block

**FIGURE 2:**
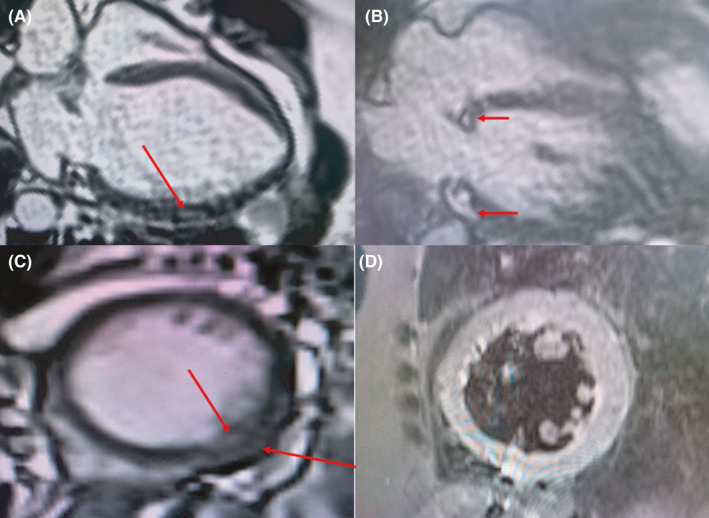
Cardiac MRI images showing patchy late gadolinium enhancement of the basal septum and mid to apical lateral LV wall A, basal septum and basal lateral wall B, and mid‐inferolateral wall C. The absence of edema or inflammation is shown in STIR images D, after treatment

A previously well 58‐year‐old woman with an unremarkable medical history was seen at the respiratory clinic with a 6‐month history of a dry cough, easy fatigability, and progressive breathlessness on exertion. She was unaware of any precipitants and had received numerous antibiotic courses, salbutamol and budesonide inhalers, and prednisolone with mild improvement of her symptoms. Physical examination including chest auscultation was normal except for a regular tachycardia of 106 beats/min. Chest X‐ray (CXR) revealed a bilateral hilar lymphadenopathy and cardiomegaly. A contrast‐enhanced computed tomography (CT) scan of the chest showed bilateral reticulonodular opacities with bilateral hilar lymphadenopathy suggestive of stage II pulmonary sarcoidosis. An electrocardiogram (ECG) showed sinus tachycardia with a left bundle branch block (Figure 1), and a transthoracic echocardiogram revealed a dilated left ventricle with severely reduced LV systolic function (ejection fraction of 30%) and dyssynchrony of the interventricular septum (IVS). Laboratory investigations showed elevated serum angiotensin‐converting enzyme (ACE) of 69.6 UI/L (8.0‐52.0), erythrocyte sedimentation rate (ESR) of 33 mm fall/hr (3.0‐5.0), and hypercalcemia of 2.79 mmol/L (2.12‐2.62). ANA was negative, and sputum for GeneXpert (GeneXpert Dx System Version 4.8) did not detect Mycobacterium tuberculosis DNA. Lung function tests revealed a restrictive pattern with a forced expiratory volume in the first second (FEV1) of 1.44L (predicted, 2.18L), a forced vital capacity (FVC) of 1.88L (predicted, 2.51L), and a FEV1/FVC ratio of 77% (predicted, 87%). A diagnosis of pulmonary sarcoidosis was made with possible cardiac involvement and steroid therapy initiated with good improvement of symptoms within 3 months. The patient developed symptoms of heart failure (orthopnoea, paroxysmal nocturnal dyspnoea, bilateral leg swelling) 6 months later when her steroid dose was being tapered. She was admitted and managed for heart failure (NYHA Class III). Cardiac MRI done later showed a moderately dilated LV with moderately reduced ejection fraction (EF) of 40%, discoordinate movement of interventricular septum, and a thinned and hypokinetic apical anterior wall. There was no active myocardial inflammation or edema, but there was midwall late gadolinium enhancement of the basal inferior LV wall, the basal lateral LV wall with patchy near‐transmural enhancement of the apical anterior, and mid to apical lateral LV wall (Figure 2) all suggestive of cardiac sarcoid. The prednisolone dose was increased to 1 mg/kg and azathioprine added to allow for prednisolone dose tapering. She remains stable on above in addition to heart failure therapy (NYHA class I). Her ECG findings remain unchanged.

### 2.2 Case 2

**FIGURE 3:**
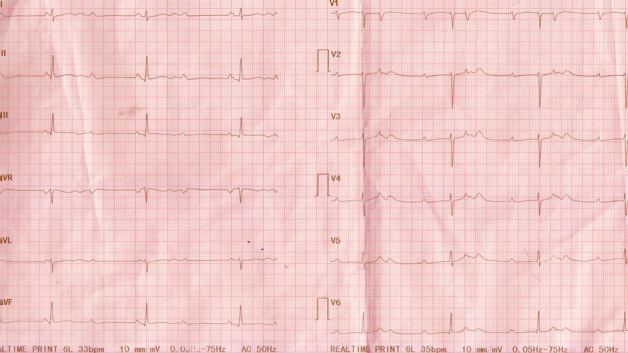
Complete heart block

**FIGURE 4:**
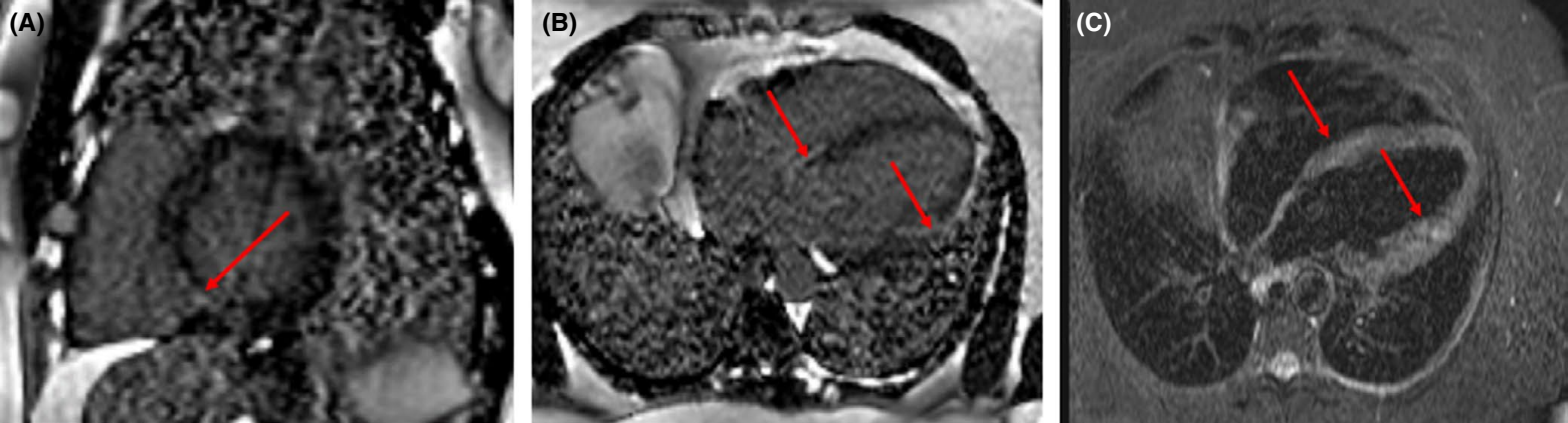
Cardiac MRI images showing late gadolinium enhancement of the mid‐septum A, basal to mid‐septum and lateral LV wall B, with corresponding edema and inflammation in STIR images C

A 51‐year‐old man, with no chronic illnesses, was seen with a 4‐month history of intermittent dry cough, palpitations, and progressive shortness of breath on exertion. He had received treatment for recurrent chest infections and was a lifelong nonsmoker. He was referred to the respiratory clinic with an abnormal CXR and a negative sputum for GeneXpert (GeneXpert Dx System Version 4.8). Clinical examination revealed bilateral lower lung zone crepitations, SpO_2_ of 94% at rest and on room air with exertional drop to 88%. He had a regular bradycardia of 35 beats/min and blood pressure of 137/74 mmHg. ECG revealed a third‐degree heart block (Figure 3). His transthoracic echocardiogram showed normal LV morphology (relative wall thickness, 0.32; LV mass index, 91 g/m^2^), normal systolic and diastolic function, and normal segmental wall motion. There was complete atrioventricular block with junctional escape rhythm as well as intermittent Mobitz type I or 2 block on his 24‐hour Holter ECG. A high‐resolution chest CT scan showed ground glass opacification with multiple tiny nodules in the mid‐ and lower lung zones, increased reticulation, and mediastinal lymphadenopathy suggestive of stage II sarcoidosis. Serum ACE was elevated; 116.0 IU/L (8.0‐52.0) and pulmonary function test showed a restrictive pattern with a FEV1 of 2.23L (predicted, 3.64), FVC of 2.53L (predicted, 4.68), and a FEV1/FVC ratio of 88.3% (predicted, 77.7%). A diagnosis of pulmonary sarcoidosis with probable cardiac involvement was made and confirmed by cardiac MRI which revealed late gadolinium enhancement of the mid‐septum, basal to mid‐septum, and lateral LV wall with corresponding edema and inflammation in STIR images highly suggestive of cardiac sarcoidosis (Figure 4).

**FIGURE 5:**
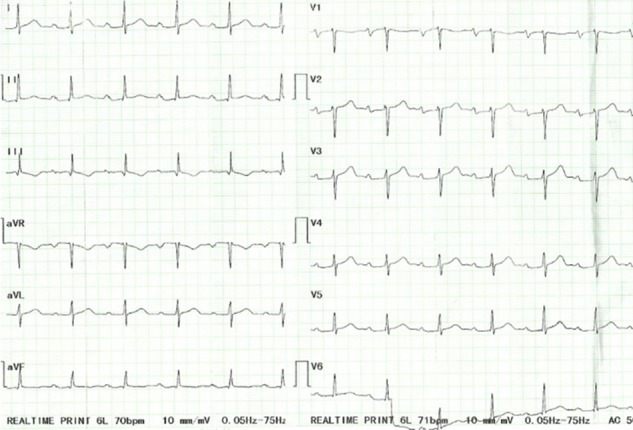
First‐degree heart block

Patient was started on corticosteroid and azathioprine and assessed for a possible pacemaker insertion which patient refused. He however showed marked improvement in the respiratory symptoms and a conversion of the complete heart block to a first‐degree heart block after 8 weeks of therapy (Figure 5). He is being followed up periodically with ECGs.

## 3 DISCUSSION

Cardiac sarcoidosis is the most ominous manifestation of sarcoidosis with a prevalence of 20 to 30% from autopsy studies as well as screening of known sarcoid patients with cardiac MRI.^2^ However, only 5% of these patients had clinical evidence of the disease.^4^, ^5^, ^10^

Although isolated CS is thought to constitute about 27%‐54% of all CS cases, recent studies involving whole‐body PET scans present lower and varying prevalence.^11^, ^12^ Hence, extensive evaluation is required to exclude evidence of sarcoid lesions in other organs prior to diagnosing isolated disease.^12^, ^13^, ^14^ Data on CS in Africa as a whole are scanty with no case reports identified.

The wide spectrum of clinical manifestations of CS arise from the variable locations of sarcoid lesions in the myocardium.^5^ There is however a predilection for the base of the interventricular septum, the conduction system, and the left ventricular (LV) free wall.^4^, ^15^, ^16^ In consistence, both presented cases had involvement of the base of the interventricular septum, the conduction system, and the left ventricular (LV) free wall.

Conduction abnormalities as observed in our report are the most occurring electrophysiological manifestation of CS with a prevalence up to 62%.^17^ Complete heart block and bundle branch blocks have been reported in 23%‐30% and 12%‐32%, respectively, in CS patients.^16^ Our first case had left bundle branch block (LBBB) which is known to be less common compared to right bundle branch blocks.^16^, ^18^, ^19^ Schuller et al found that among a cohort of patients with biopsy‐proven pulmonary sarcoidosis, a bundle branch block pattern is associated with cardiac involvement.^18^

Left ventricular systolic and diastolic dysfunction leading to progressive congestive heart failure often results from conduction or rhythmic abnormalities, extensive myocardial infiltration, or both.^16^, ^20^, ^21^ In our first case, the noted left ventricular dysfunction was due to the LBBB pattern and myocardial infiltration seen on the cardiac MRI.

Studies have identified previously undiagnosed CS in explanted hearts that had a clinical diagnosis of nonischemic cardiomyopathy and in patients with advanced heart failure requiring LV assist device implantation.^22^, ^23^

The diagnosis of CS requires suggestive clinical features, presence of noncaseating myocardial granulomas, and the exclusion of alternative causes of granulomatous disease.^24^, ^25^, ^26^ Several established diagnostic guidelines in existence are based on expert consensus with none validated by prospective data or clinical trials.^27^

The current American Thoracic Society guidelines recommend screening for CS using patient symptoms or signs and, where suspected to have CS, advise cardiac MRI and/or PET as initial investigation to obtain diagnostic and prognostic information.^24^ In our cases, the signs at presentation were tachycardia and bradycardia with ECG evidence of sinus tachycardia/LBBB and complete heart block, respectively. Both patients subsequently had cardiac MRI.

The Heart Rhythm Society (HRS) has two diagnostic pathways: (a) histological evidence of noncaseating granulomas in myocardium and (b) clinical diagnosis (histological evidence of extracardiac sarcoidosis plus one or more of the following: steroid± immunosuppressant responsive cardiomyopathy or heart block, unexplained EF <40%, unexplained VT, second/third‐degree AVB, patchy uptake of FDG‐PET, LGE on MRI and positive gallium uptake consistent with CS).^26^

The gold standard for diagnosing CS is endomyocardial biopsy (EMB), but the diagnostic yield has historically been low with a sensitivity of 10%‐25% using fluoroscopy‐guided but nontargeted right ventricular biopsy practiced in the 1980s and 1990s.^28^, ^29^, ^30^ However, the yield of EMB has improved with the help of modern cardiac imaging and electroanatomic mapping coupled with lower risk of serious complications (<1%) in experienced hands.^11^, ^31^, ^32^ In the absence of EMB, the presence of unexplained left ventricular dysfunction with EF <40% and heart block being responsive to steroids in both cases as well as evidence of LGE in a typical pattern on MRI made the diagnosis of CS highly probable in our patients.^24^, ^25^, ^26^ The presence of cardiac complications, however, prevented biopsy of the lung for histological confirmation of extracardiac sarcoidosis. Chest HRCT, which has shown significant correlation with histological features of pulmonary sarcoidosis, was suggestive of lung involvement in both cases.^33^ Laboratory markers such as serum ACE and hypercalcemia further supported the diagnosis of pulmonary sarcoidosis. ^33^

Cardiac MRI plays a significant role because it provides a noninvasive means of detecting morphological and functional abnormalities consistent with CS.^4^, ^17^, ^34^, ^35^ In addition to its high sensitivity and specificity, it comes handy in areas where EMB is unavailable.^34^, ^35^ Fluorodeoxyglucose‐positron emission tomography (FDG‐PET) scan is another useful nuclear imaging modality that employs radioactive glucose in detecting areas of active inflammation in CS with a sensitivity and specificity of 89% and 78%, respectively.^36^ However, FDG‐PET is even less accessible in many resource‐limited countries.

Systemic corticosteroids remain the cornerstone of treatment in CS and are considered first‐line agents because of their efficacy and attainment of significant response over a relatively short period. Nonsteroidal immunosuppressive drugs such as methotrexate, azathioprine, mycophenolate mofetil, leflunomide, cyclosporine, or cyclophosphamide are alternatives in the event of corticosteroids failure and development of adverse effects, particularly when higher doses are required (>10 mg/day prednisolone).^4^, ^9^, ^37^

TNF‐alpha inhibitors (infliximab and adalimumab) have recently proven effective in steroid/nonsteroidal immunosuppressant refractory cases.^38^, ^39^ In a cohort of 36 CS patients evaluated by Harper et al, use of infliximab resulted in lower steroid doses and less dysrhythmia.^40^

Since heart rhythm disorders frequently cause death in CS patients, implantable cardiac defibrillators (ICDs) play a prominent role in its managment.^8^ It also reduces the risk of arrhythmic SCD which is highest in patients with LGE on MRI, history of sustained ventricular arrhythmia, inducible ventricular arrhythmias during electrophysiological studies (EPS), and left ventricular dysfunction.^41^, ^42^

Current guidelines therefore recommend ICD implantation in CS patients with sustained VT, post cardiac arrest, and LVEF of 35% or less. Patients with LVEF >35% who have had syncope, evidence of myocardial scar on cardiac MRI or PET scan, indication for permanent pacing, and/or inducible ventricular arrhythmias during EPS are candidates for ICD implantation. ^26^, ^43^, ^44^ Although both cases required ICD, this was not readily available so a pacemaker was suggested for case 2 which he refused.

Guideline‐directed medical management of heart failure as with other aetiologies is also recommended.^9^ Antiarrhythmic agents including amiodarone may be used to maintain sinus rhythm in patients with atrial arrhythmias or symptomatic VT.^9^

In patients with VT refractory to medical therapy, catheter ablation, an effective management option, helps with substantial reduction of total arrhythmia burden.^17^, ^36^, ^42^ Cardiac transplantation as a last resort should be considered in CS patients with intractable ventricular arrhythmias or end‐stage heart failure.^28^

## 4 CONCLUSION

Diagnosing CS requires a high index of suspicion especially in patients with systemic sarcoidosis. Active screening of such patients will promote early diagnosis and treatment which can be lifesaving.

## CONFLICT OF INTEREST

No conflict of interest.

## AUTHOR CONTRIBUTIONS

**Felix Razak Awindaogo and Jane Sandra Afriyie‐Mensah:** conceptualized the study and wrote, reviewed, and edited the manuscript. **Harold Ayetey:** wrote, reviewed, and edited the manuscript and analyzed and interpreted the cardiac MRI images. **Emmanuella Naa Deedei Tagoe:** wrote, reviewed, and edited the manuscript. All authors were involved in editing and approval of the manuscript.

## ETHICAL APPROVAL

Consent obtained from patients.

## Data Availability

All data included in this report are accurate to the best of our knowledge. We will make available data (images and reports) upon request.
